# Genotypic prevalence of norovirus GII in gastroenteritis outpatients in Shanghai from 2016 to 2018

**DOI:** 10.1186/s13099-019-0321-x

**Published:** 2019-07-26

**Authors:** Xiaozhou Kuang, Zheng Teng, Xi Zhang

**Affiliations:** Shanghai Municipal Center for Disease Control and Prevention, 1380 Zhongshan Road (west), Shanghai, 200336 China

**Keywords:** Norovirus GII, Gastroenteritis outpatients, Genotype, Recombinant strain

## Abstract

**Background:**

With the help of an existing citywide comprehensive surveillance on gastroenteritis outpatients, although norovirus genogroup II (NoV GII) was tested routinely, its genotypes were never investigated systematically on a municipal level. This study aimed to understand the prevalence, major genotypes and evolutional trends of NoV GII in Shanghai during the period of 2016–2018, and to provide molecular bases for early warning for any potential NoV outbreaks.

**Methods:**

27 sentinel hospitals from all 16 districts were recruited by stratified probability proportional to size (PPS) method in Shanghai comprehensive diarrhea surveillance programme. Stool samples were collected and screened for NoV GII by real-time reverse transcription polymerase chain reaction (qRT-PCR). For samples that were positive in qRT-PCR, conventional RT-PCR was performed to amplify the ORF1-ORF2 junction of NoV GII gene. Generated sequences were typed by RIVM online genotyping tool, and then strains of interest were analyzed phylogenetically using MEGA 6.0.

**Results:**

A total of 7883 stool samples were collected from diarrhea outpatients, among which 6474 were from adults and 1409 were from children. 13.66% (1077 cases) were screened positive in qRT-PCR for NoV GII, from which 71.96% (775 cases) were sequenced successfully. The top three genotypes were GII.Pe/GII.4 (37%), GII.P17/GII.17 (26%) and GII.P16/GII.2 (17%). While GII.Pe/GII.4 detection rate decreased significantly over the 3 years (from 48.4 to 20.9%); GII.P16/GII.2 appeared for the first time in October 2016 and rose rapidly to 27.0% in 2017, but fell back to 23.4% in 2018. Meanwhile there was a significant increase for both GII.P12/GII.3 and GII.P7/GII.6 recombinant genotypes detected in adult population in 2018. Phylogenic analysis revealed the existence of multiple gene clusters within both of these recombinant genotypes.

**Conclusion:**

Unlike the alternating circulation of GII.4 and non-GII.4 NoV observed in 2016 or 2017, the genotype profile of NoV GII in 2018 was characterized by the co-prevalence of multiple recombinant genotypes. A recent increase in detection rate in less reported recombinant genotypes such as GII.P12/GII.3 and GII.P7/GII.6 among adult population calls for a continuing close monitoring on NoV GII genotypes in case of potential local outbreaks.

**Electronic supplementary material:**

The online version of this article (10.1186/s13099-019-0321-x) contains supplementary material, which is available to authorized users.

## Background

Norovirus (NoV) is the leading cause of acute gastroenteritis worldwide. All age groups are susceptible to its infection, and its detection rate ranks first in both sporadic cases and outbreaks [1–3]. The economic burden attributed to NoV infection on the health care system is about $4.2 billion, and the burden on social economy is as high as $60.3 billion [4]. Previous comprehensive monitoring of gastroenteritis outpatients in Shanghai had revealed that NoV was found in ~ 60% of all diarrhea outpatients [5], among which NoV GII accounted for 90% of all NoV infections [6].

In terms of its molecular evolution, NoV is susceptible to rapid mutations through gene recombination, which is commonly observed in its ORF1-ORF2 junction [7]. In China, GII.Pe/GII.4 Sydney strain first appeared in 2012 [8], then a new GII.17 strain was found during the winter of 2014 [9], subsequently a recombinant GII.P16/GII.2 genotype emerged at the end of 2016 [10]. In Shanghai, GII.Pe/GII.4 Sydney strain was first reported In September 2012 [11], but its dominance was gradually replaced by GII.P17/GII.17 in 2015 [12]. Up until 2016,the circulation of local NoV genotypes was similar to that reported nationwide, and around the world [13–15]. This study aimed to ascertain the time of first appearance of local GII.P16/GII.2 strain after 2016, and to describe the prevalence and circulation of other genotypes in the past 3 years including GII.P12/GII.3 and GII.P7/GII.6, which were increasingly reported recently worldwide in outbreaks [16–18]. Finding in this study could provide scientific basis for the early warning of potential NoV GII outbreaks.

## Methods

### Case definition

Outpatients who sought medical care at the sentinel clinics, with a daily bowel movements 3 times or more, accompanied by loose or liquid stools [the definition of diarrhea by the World Health Organization (WHO)] [19].

### Sentinel site recruitment and case sampling

Sentinel recruitment for comprehensive surveillance of diarrheal diseases programme in Shanghai were detailed in previous literature [6]: briefly, all hospitals containing enteric clinics in Shanghai were divided into urban and suburban groups, and then each group was further stratified into primary, secondary and tertiary hospital subgroups, and finally 27 hospitals are drawn by this stratified PPS method, covering 22 adults and 5 pediatric sentinel sites. Adult enteric clinics recruited outpatients over the age of 14, while pediatric clinics recruited outpatients under 14-year according to the definition of these two types of clinics in China. Diarrhea cases were sampled at pre-estimated sampling intervals in each sentinel clinic.

### Specimen collection and storage

5 g of stool was collected into a plastic cup without the addition of any chemical substances on the day of patient’s hospital visit. It was then temporarily stored at 4 °C at the sentinel site and delivered in an ice-packed biosafety transport carrier to the laboratory on the same day.

### Sample process prior to RNA Extraction

5% saline solution was added to the sample to make a 10% fecal suspensions, it was then thoroughly vortexed, aliquoted and then centrifuged at ×8000*g* for 5 min for immediate testing.

### RNA extraction

200 μl of the centrifuged supernatant was aspirated, and extracted using Roche MagNA Pure LC 2.0 extractor with Roche Total Nucleic Acid Isolation Kit (Roche Applied Science, Switzerland) according to the manufacturer’s instructions.

### qRT-PCR screening

All specimens were double screened for NoV GII by qRT-PCR using commercially available kits (Shanghai Zhijiang Biotechnology Co., Ltd. and Jiangsu Shuoshi Biotechnology Co., Ltd.). Both kits targeted the ORF1-ORF2 junction of NoV GII gene.

### NoV GII genotyping

For samples that were positive in both qRT-PCR kits, NoV GII ORF1-ORF2 junction (also known as the polymerase-capsid region) was amplified using method designed by the US CDC [20]. QIAxcel capillary electrophoresis was run with QIAxcel DNA Screening Kit (Qiagen, Hilden, Germany) to determine whether the specimen was successfully amplified and showed an expectant product size of 570 bp. All specimens positive with the target fragment size were sequenced by ABI sequencer 3730 DNA analyzer with BigDye™ Terminator v3.1 kit (Applied Biosystems, California, US). The resulting sequences were spliced using Sequncher software v4.1.4 (Gene Codes, US) [21] and then genotyped by RIVM online Norovirus genotyping tool (http://www.rivm.nl/mpf/norovirus/typingtool,RIVM,MA Bilthoven, Netherlands). Sequences representative of the main variants of the recombinant strains focused in this study were deposited in GenBank (accession numbers MK779279-MK779304; MK789447–MK789463—Additional file data) (Additional file 1).

### Phylogenic analysis

For strains of interest, phylogenetic trees were constructed using the neighbour-joining method in MEGA version 6.0 software. Sequences were aligned using Clustal W with the Kimura-2 parameter. Tree robustness was determined by bootstrapping using 1000 pseudo replicates [22].

### Statistical analysis

All calculations were conducted using Microsoft Excel 2010 and SPSS software v16.0 (IBM, USA) where Pearson Chi square test or the Fisher’s exact with two-tailed method was used to determine statistical significance with P < 0.05.

## Results

### NoV GII positive detection rate

From January 2016 to December 2018, a total of 7883 stool samples were collected from diarrhea outpatients, including 2896 samples from 2016, 2622 samples in 2017, and 2365 samples from 2018. A total of 1077 samples (13.66%) were positive for NoV GII by qRT-PCR (Table 1). NoV GII screen positive rate in adults was significantly higher than that in children (χ^2^ = 8.725, P < 0.05).

**Table 1:** NoV GII qRT-PCR screening results in adult and children outpatients in 2016–2018

Year	Adults				Children			
	No. of cases	Screen POS no.	Screen POS rate (%)	χ^2^ (P-value)	No. of cases	Screen POS no.	Screen POS rate (%)	χ^2^ (P-value)
2016	2443	339	13.88	18.02 (< 0.05)	453	50	11.04	0.58 (= 0.75)
2017	2123	352	16.58		499	60	12.02	
2018	1908	228	11.95		457	48	10.50	
Total	6474	919	14.20		1409	158	11.21	

### Seasonal variation in NoV GII detection rate

**Fig. 1:**
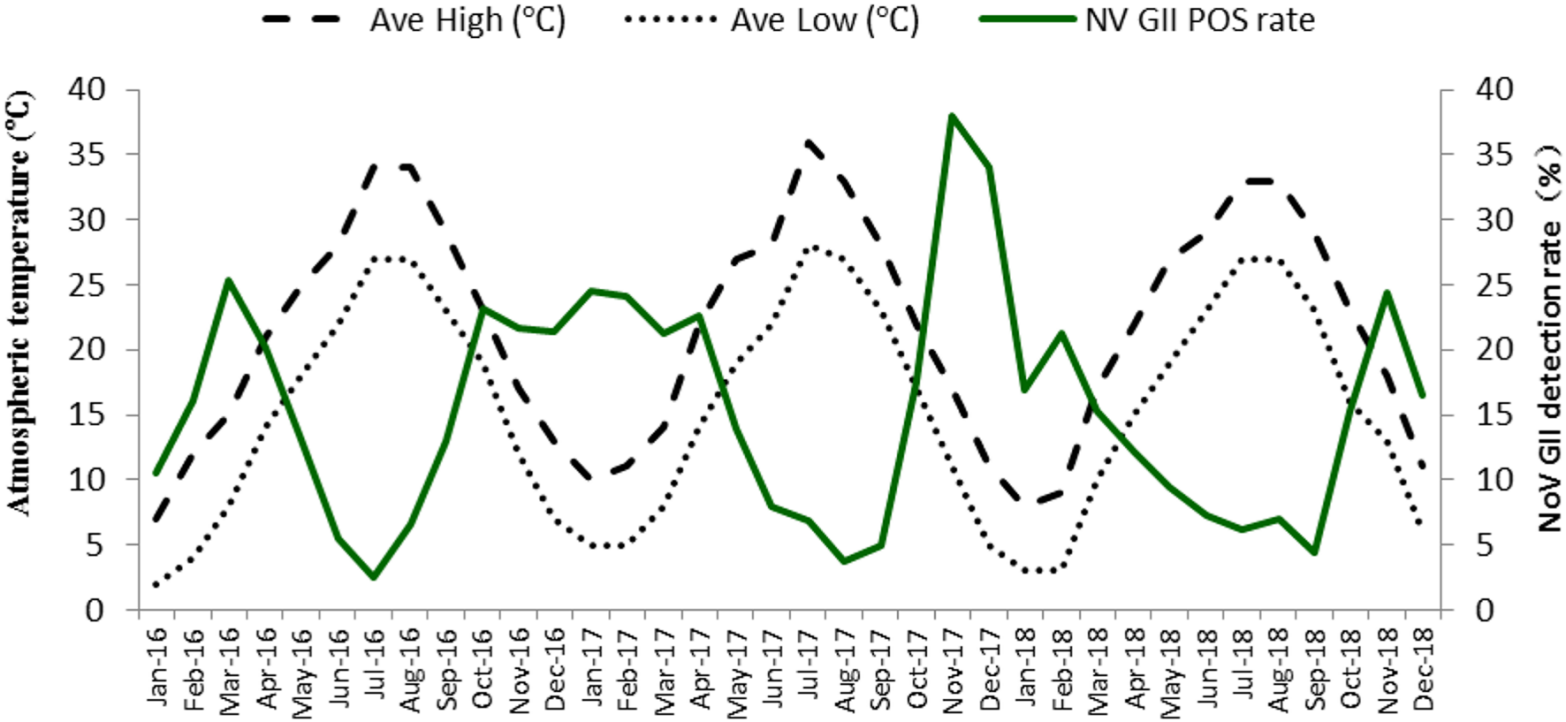
Changes in the positive detection rate of NoV GII at different times and atmospheric temperatures in Shanghai during 2016–2018

The positive detection rate generally peaked from autumn/winter to the next spring. It reached a historical apex at 37.93% in the winter of 2017 (Fig. 1). On the contrary, when the average atmospheric temperature was highest in the summers, NoV GII detection rate plummeted as shown in Fig. 1.

### NoV ORF1-ORF2 (polymerase-capsid region) genotypes overall constitution

Of 1077 NoV GII qRT-PCR positive samples, 775 were successfully sequenced. A total of 16 genotypes were obtained, of which, five of the most prominent genotypes were GII.Pe/GII.4, GII.P17/GII.17, GII.P16/GII.2, GII.P12/GII.3 and GII.P7/GII.6. Among these, 71.61% (555 samples) were recombinant genotypes (Table 2). No co-infection by different NoV GII genotypes was discovered in these samples.

**Table 2:** Compositional percentage of NoV GII genotypes found in 2016–2018

Polymerase-capsid region genotype	No. of samples	Compositional percentage (%)
GII.Pe/GII.4	288	37.16
GII.P17/GII.17	198	25.55
GII.P16/GII.2	131	16.90
GII.P12/GII.3	53	6.84
GII.P7/GII.6	27	3.48
GII.P16/GII.13	23	2.97
GII.P21/GII.21	11	1.42
GII.Pe/GII.17	10	1.29
GII.P16/GII.4	8	1.03
GII.P8/GII.8	6	0.77
GII.P21/GII.13	6	0.77
GII.P15/GII.15	5	0.65
GII.P22/GII.5	4	0.52
GII.Pg/GII.1	3	0.39
GII.P7/GII.14	1	0.13
GII.P7/GII.9	1	0.13
Total	775	100

### Time distribution of main genotypes

**Fig. 2:**
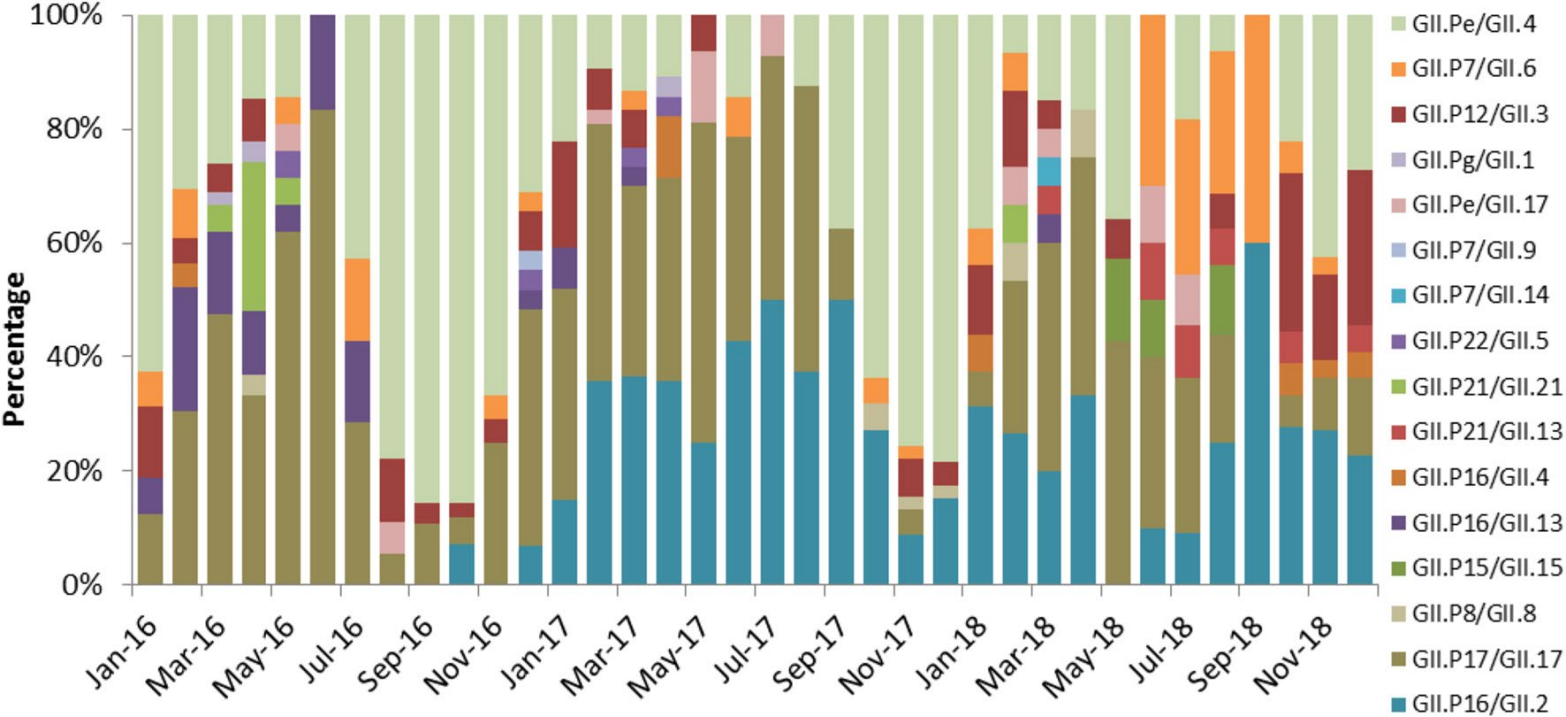
Time distribution of different NoV GII genotypes from 2016 to 2018

For the predominant GII.Pe/GII.4 genotype, its proportion in January and August-to-November of 2016 as well as in October-to-December of 2017 exceeded the total proportion of all other genotypes at that time (Fig. 2). Moreover the prevalence of GII.Pe/GII.4 declined over 3 years (Table 3). Meanwhile although the second most prominent genotype GII.P17/GII.17 was prevalent throughout the year and remained relatively stable over the whole period, its percentage dropped to the lowest in August-to-October of 2016 and winter in 2017. It is worth noting that GII.P16/GII.2, which ranked third in prevalence, first appeared in October 2016 and continued to rise in 2017 and become the dominant genotype along with GII.Pe/GII.4 and GII.P17/GII.17. On the other hand, by 2018, the proportion of GII.P12/GII.3 and GII.P7/GII.6 had gradually increased, and the peak of GII.P7/GII.6 appeared around the summer of 2018, while the peak of GII.P12/GII.3 appeared in October-December 2018 (Table 3, Fig. 2).

**Table 3:** Annual change in compositional percentage of main genotypes

Genotype	Number/Percentage (%)				
	2016	2017	2018	Total	χ^2^ (P-value)
GII.Pe/GII.4	137 (48.41%)	107 (35.67%)	44 (22.92%)	288 (37.16%)	32.31 (< 0.05)
GII.P17/GII.17	82 (28.98%)	76 (25.33%)	40 (20.83%)	198 (25.55%)	4.00 (= 0.14)
GII.P16/GII.2	5 (1.77%)	81 (27.00%)	45 (23.44%)	131 (16.90%)	73.77 (< 0.05)
GII.P12/GII.3	14 (4.95%)	16 (5.33%)	23 (11.98%)	53 (6.84%)	10.62 (< 0.05)
GII.P7/GII.6	7 (2.47%)	4 (1.33%)	16 (8.33%)	27 (3.48%)	18.41 (< 0.05)
Others	38 (13.43%)	16 (5.33%)	24 (12.5%)	78 (10.06%)	12.21 (< 0.05)
Total	283 (36.52%)	300 (38.71%)	192 (24.77%)	775 (100.00%)	

### Comparison of NoV GII genotypes found in adults and children outpatients

658 sequences were obtained from adult cases, which can be further divided into 16 genotypes. The five most prevalent types were GII.Pe/GII.4 (214 cases, 32.52%), GII.P17/GII.17 (194 cases, 29.48%), GII.P16/GII.2 (110 cases, 16.72%), GII.P12/GII.3 (41 cases, 6.23%), GII.P7/GII.6 (24 cases, 3.65%) (Table 4). The remaining genotypes include GII.P16/GII.13 (23 cases, 3.50%), GII.Pe/GII.17 and GII.P21/GII.21 (10 cases each, 1.52%), GII.P8/GII. 8, GII.P16/GII.4 and GII.P21/GII.13 (6 cases each, 0.91%), GII.P15/GII.15 (5 cases, 0.76%), GII.P22/GII.5 (4 cases, 0.61%), GII.Pg/GII.1 (3 cases, 0.46%), GII.P7/GII.14 and GII.P7/GII.9 (1 case each, 0.15%).

**Table 4:** The annual percentage of the top 5 genotypes found in both adults and children

Population	Year	GII.Pe/GII.4		GII.P17/GII.17		GII.P16/GII.2		GII.P12/GII.3		GII.P7/GII.6		Other genotypes		Total no.
		No./(rate %)	χ^2^ (P-value)	No./(rate %)	χ^2^ (P-value)	No./(rate %)	χ^2^ (P-value)	No./(rate %)	χ^2^ (P-value)	No./(rate %)	χ^2^ (P-value)	No./(rate %)	χ^2^ (P-value)	
Adults	2016	102 (41.98)	19.16 (< 0.05)	81 (33.33)	3.54 (= 0.18)	5 (2.06)	66.78 (< 0.05)	13 (5.35)	9.94 (< 0.05)	6 (2.47)	16.38 (< 0.05)	36 (14.81)	11.18 (< 0.05)	243
	2017	78 (30.35)		74 (28.79)		75 (29.18)		10 (3.89)		4 (1.56)		16 (6.23)		257
	2018	34 (21.52)		39 (24.68)		30 (18.98)		18 (11.39)		14 (8.86)		23 (14.56)		158
Children	2016	35 (87.50)	36.91 (< 0.05)	1 (2.50)	0.32 (= 1.00)	0 (0.00)	25.02 (< 0.05)	1 (2.50)	3.98 (= 0.14)	1 (2.50)	1.20 (= 0.53)	2 (5.00)	2.10 (= 0.39)	40
	2017	29 (67.44)		2 (4.65)		6 (13.95)		6 (13.95)		0 (0.00)		0 (0.00)		43
	2018	10 (29.41)		1 (2.94)		15 (18.09)		5 (14.71)		2 (5.88)		1 (2.94)		34
Adults	Total	214 (32.52)	40.16 (< 0.05)	194 (29.48)	35.48 (< 0.05)	110 (16.72)	0.11 (= 0.79)	41 (6.23)	2.53 (= 0.16)	24 (3.65)	0.35 (= 0.61)	75 (11.40)	8.57 (< 0.05)	658
Children		74 (63.25)		4 (3.42)		21 (17.95)		12 (10.26)		3 (2.56)		3 (2.56)		117

Meanwhile, 117 sequences were obtained from children’s cases, which can be further divided into 7 genotypes. GII.Pe/GII.4 accounted for the highest proportion (74 cases, 63.25%), it is followed by GII.P16/GII.2 (21 cases, 17.95%), GII.P12/GII.3 (12 cases, 10.26%), GII.P17/GII.17 (4 cases, 3.42%), GII.P7/GII.6 (3 cases, 2.56%), GII.P16/GII.4 (2 cases, 1.71%) and GII.P21/GII.21 (1 case, 0.85%) Table 4 summarized the above finding.

**Fig. 3:**
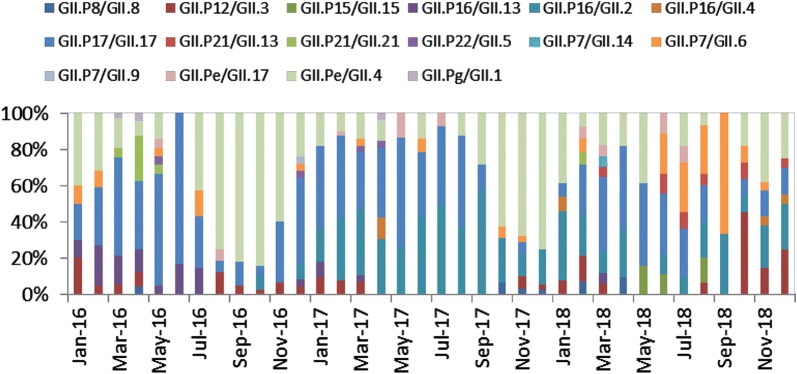
Time distribution of different genotypes of NoV GII in adults

**Fig. 4:**
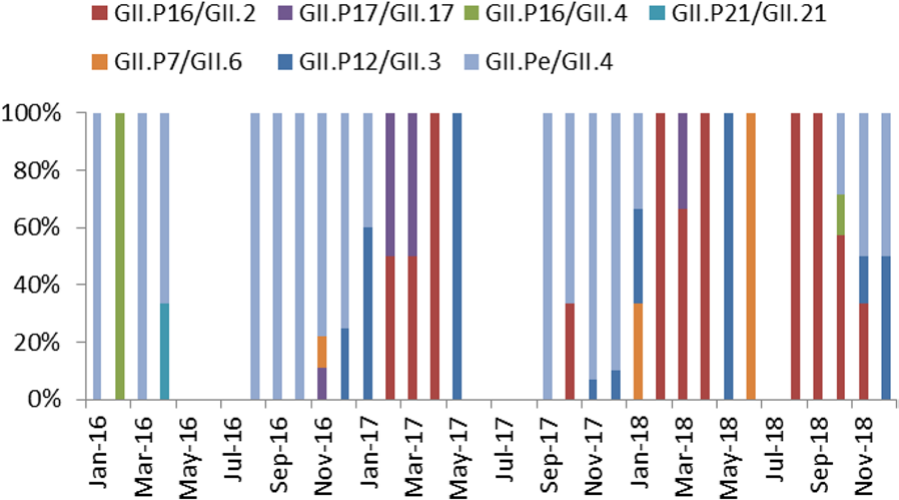
Time distribution of different genotypes of NoV GII in children

The percentage of GII.Pe/GII.4 in both adults and children declined significantly over the years, and its prevalence in children was higher than that in adults. GII.P16/GII.2 underwent a significant change over 3 years in both adults and children, but no overall difference in its prevalence was observed between adults and children. The prevalence of GII.P17/GII.17 stayed relatively stable in both populations throughout the period, but its prevalence in adults was higher than that in children. While the prevalence of GII.P12/GII.3 and GII.P7/GII.6 in children remained stable over 3 years, significant changes was detected in adults for both strains (Table 4). Figures 3 and 4 shows the time distribution of all genotypes in both populations.

### Partial genetic analysis of less reported genotypes GII.P12/GII.3 and GII.P7/GII.6 that displayed a significant rise recently in adults

**Fig. 5:**
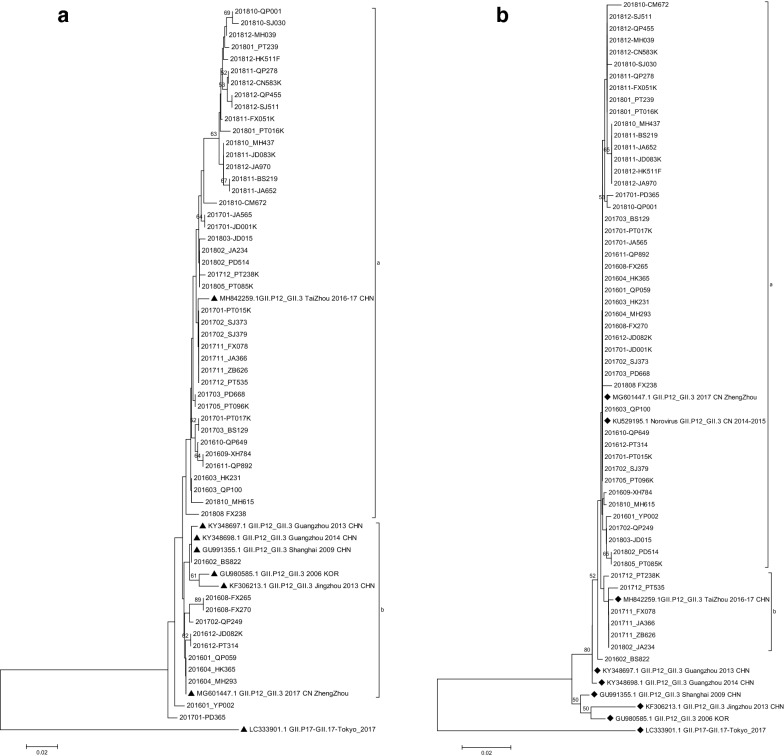
Phylogenic analysis of NoV GII.P12/GII.3 (**a**) partial ORF1 gene (RdRp 273 bp, nt 4832-5104 according to reference strain: MG601447) with reference strains shown in black triangles (**b**) partial ORF2 gene (Capsid 282 bp, nt 5085-5366 according to reference strain: MG601447) with reference strains shown in black diamonds. The trees were constructed in Mega 6.0 through the Neighbor joining method. The bootstrap values generated from 1000 replicates are shown at nodes, and only bootstrap values > 50% are shown

NoV GII.P12/GII.3 phylogenic analysis showed that its polymerase region is basically divided into two clusters, one group clustered with reference strains reported in China from 2009 to 2017 and in Korea from 2006. Among these, strains isolated in this study from 2016 accounted for 11.9% (5/42), strains isolated from 2017 accounted for 33.3% (14/42) and strains isolated from 2018 accounted for 54.8% (23/42) of all. A second group clustered with reference strain found in Zhengzhou in 2017. Among these, local strains isolated from 2016 occupied 88.9% (8/9) and strain isolated from 2017 occupied 11.1% (1/9). Additional, there were two lone strains which belong to neither of these two clusters (Fig. 5a).

**Fig. 6:**
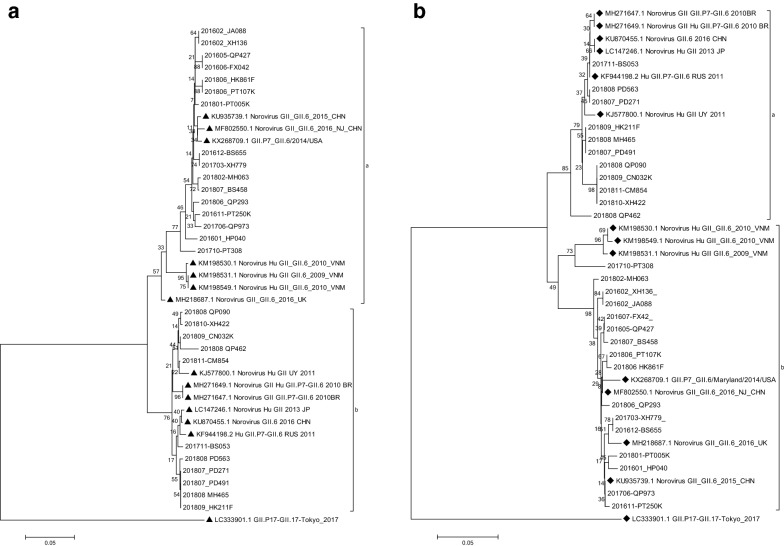
Phylogenic analysis of NoV GII.P7/GII.6 (**a**) partial ORF1 gene (RdRp 276 bp, nt 4815-5090 according to reference strain: KX268709) with reference strains shown in black triangles (**b**) partial ORF2 gene (Capsid 282 bp, nt 5074-5361 according to reference strain: KX268709) with reference strains shown in black diamonds. The trees were constructed in Mega 6.0 through the Neighbor joining method. The bootstrap values generated from 1000 replicates are shown at nodes, and only bootstrap values > 50% are shown

Phylogenic tree of partial ORF2 gene of GII.P12/GII.3 is also divided into two clusters. One group clustered with 2017 Zhengzhou reference strain and other reference strains reported in China from 2014 to 2015. Among these, local strain isolated from 2016 accounted for 28.3%. (13/46), strains isolated from 2017 accounted for 23.9% (11/46) and strains isolated from 2018 accounted for 47.8% (22/46) of all. A second group clustered with Taizhou 2016–2017 reference strain. Among these, local strains isolated from 2017 occupied 83.3% (5/6), strains isolated from 2018 occupied 16.7% (1/6). There was only one lone strain which cannot be classified into either of two clusters (Fig. 5b).

NoV GII.P7/GII.6 phylogenic analysis showed that its polymerase region is basically divided into two clusters, one group clustered with reference strains reported from China during 2015–2016, from Vietnam during 2009–2010, from 2014 US and 2016 UK. Among these, strains isolated in this study from 2016 accounted for 43.75% (7/16), strains isolated from 2017 accounted for 18.75% (3/16) and strains isolated from 2018 accounted for 37.5% (6/16) of all. A second group clustered with reference strain found in 2010 Brazil, 2011 Uruguay, 2011 Russia, 2013 Japan and 2016 China. Among these, local strains isolated from 2017 occupied 9.1% (1/11) and strains isolated from 2018 occupied 90.9% (10/11) (Fig. 6a).

Phylogenic tree of partial ORF2 gene of GII.P7/GII.6 is also divided into two clusters. One group clustered with 2010 Brazil, 2011 Uruguay, 2011 Russia, 2013 Japan and 2016 China. Among these, local strain isolated from 2017 accounted for 9.09% (1/11) and strains isolated from 2018 accounted for 90.91% (10/11) of all. A second group clustered with reference strains reported from China during 2015–2016, from Vietnam during 2009–2010, from 2014 US and 2016 UK. Among these, local strains isolated from 2016 occupied 43.75% (7/16), strains isolated from 2017 occupied 18.75% (3/16), strains isolated from 2018 occupied 37.50% (6/16) (Fig. 6b).

## Discussion

### NoV detection rate

The average detection rate of NoV GII (13.66%) was slightly higher than 10.43% reported in the diarrhea outpatients surveillance carried out in 27 provinces in 2009–2013 [23]; it was also higher than 10.50% described in study in southwestern province during 2014–2015 [24]. As only conventional RT-PCR was used in these researches, a higher NoV detection rate in this study might be attributed to a more sensitive qRT-PCR method. Although NoV GII detection rate in children was comparable to that in equivalent population in the neighboring city of Nanjing during 2017–2018 [25], it is significantly lower than that in adults. This might be caused by the large difference in sampling size between two populations.

### Seasonal changes in NoV GII infection rate

The seasonal characteristics of local NoV GII were similar to that depicted in other provinces in China [23, 24], with its peak detection rate appeared in autumn/winter to early spring, and its lowest detection rate in summer. A 10-year study in Hong Kong suggested that high atmospheric temperatures were statistically associated with low hospital admission rates caused by NoV infection, and that high relative humidity was also associated with high hospital admission rates [26]. Since this study lacks information on the temperature and relative humidity at the onset of each case, it is not possible to verify the Hong Kong study from a statistical point of view, but the general changing pattern of NoV positive rate over temperature change seems to be consistent with the finding in Hong Kong.

Evidence from other studies has demonstrated that with the increase in temperature, norovirus lost its viability and infectivity rapidly [27, 28]. Therefore NoV seems to be more stable under cold climate and thus gets transmitted more easily among people. Another possible factor that might contribute to the rise of NoV in cold season is consumption of contaminated food, such as shellfish, which is often eaten raw locally. It is through wastewater discharges that NoV enters the water system and leads to the contamination of shellfish in waters. Studies on the NoV contamination in oyster population indicated that oysters captured in cold seasons were more heavily contaminated by NoV than that captured during warm seasons [29].

All of the above might help in explaining the seasonal change of NoV infection rate in general, though the underlying mechanism for why there is an optimal condition for its replication and dissemination in human at cold seasons are still unclear. And further studies on local seafood consumption, contamination level of local seafood are needed to better understand the correlation between exposure and infection status.

### NoV GII genotypic distribution

Majority of sequenced samples were found to be recombinant genotypes. This finding confirms that recombination is an important mechanism in generating genetic diversity in NoV. This in term results in antigenic variation, which could counteract host defense system, and even affect herd immunity in its evolution; and becomes an important factor contributing to the emergence of novel NoVs in human population [7]. This progress was reflected by the finding that despite the gradual decline of GII.Pe/GII.4 over 3 years; similar decline was not observed in GII.P17/GII.17. This is possibly owing to the fact that GII.Pe/GII.4 has a longer establishment in the population than more recently emerged GII.P17/GII.17 genotype, and it takes years for the herd immunity to fight against one particular genotype.

Every 2–4 years, there would emerge a new NoV strain, quite often a recombinant strain, to replace its predecessor and gains its new dominance in circulation. GII.Pe/GII.4 Sydney strain has spread to the world and China since 2012 [3, 30, 31], and then in the winter of 2014, a new GII.P17/GII.17 strain start to emerge in Guangdong, Japan, US and other part of the world [32–34]. Another 2 years had passed before the emergence of a new recombinant GII.P16/GII.2 in the winter of 2016 throughout the world [13–15]. The prevalence of different NoV GII genotypes and the appearance of GII.P16/GII.2 recombinant strain in this study were consistent with the general evolvement of NoV GII in the rest of the world. It is also worth noting that a rise in NoV GII detection rate in 2017 coincided with the new emergence and rise of GII.P16/GII.2 recombinant genotype.

During 2016–2017, GII.4 genotype and other non-GII.4 genotypes had displayed a bimodal seasonal alternating pattern, that is, GII.4 dominated the epidemic in autumn–winter season, whereas non-GII.4 genotypes dominated the spring–summer season. These results were consistent with the finding in Hong Kong [35] although their seasonality is different from this study probably attributed to a difference in regional climate. It was proposed that this peculiar pattern might reflect a complex virus–human immunologic interaction on individual and population levels; and like in influenza virus, the mechanism itself might be used by the virus to generate antigenic novelty [35, 36]. More detailed research on why each NoV GII genotype had exhibited their distinct circulation pattern was still under progress, and should be considered for future study. However, in contrast to the bimodal pattern exhibited during 2016–2017, the prevalence pattern in 2018 was characterized by the coexistence of multiply genotypes. This change in prevalence pattern might be another mode in NoV evolution as the co-circulation provides a potential opportunity for gene exchange among different genotypes and might encourage the emergence of new strains. However further investigations are necessary for the confirmation of such hypothesis.

In addition, this study showed that the prevalence of GII.4 by the end of 2018 still remained higher than any other types, which is different from the finding in Shanghai Pudong New Area, in which the prevalence GII.P17/GII.17 has already exceeded GII.Pe/GII.4 by the middle of 2015 [37]. This discrepancy might be due to the difference in sampling techniques and area representativeness.

### Less reported recombinant genotypes such as GII.P12/GII.3 and GII.P7/GII.6 that had risen recently in adult population

Simultaneous presence of multiple gene clusters was observed in both GII.P12/GII.3 and GII.P7/GII.6 recombinants. This might provide opportunity for genetic exchange among different variants of the same genotype for the emergence of new strains. Studies have shown that GII.3 NoV had one of the highest detection rates in sporadic cases before the 1997–1990s, and its evolution rate was approximately 4.16 × 10^−3^ base substitution/site/year, which was almost as high as the more prevalent GII.4 [38]. But unlike GII.4, the selective evolutionary pressure of GII.3 is mainly driven by the host [38]. On the other hand, GII.P7/GII.6 cannot be ignored for this recombinant strain has been present in Asia and the world for at least 20 years, and has been frequently found in recent outbreaks, accompanied with a significant increase of sequence submission to GenBank [17, 18]. Therefore it is essential to carry on the routine monitoring of the above recombinant strains for their prevalence change. However for more insight into whether these gene variations in different clusters would indeed affect the binding of the virus to the host receptor or the infectivity of virus, longer sequence analysis covering complete genome and protein structure analysis will be required in future study.

## Conclusion

While an alternating predominance of GII.4 and Non-GII.4 was observed in Shanghai gastroenteritis outpatients in 2016–2017, the circulation pattern of NoV GII was less distinct in 2018 with the co-prevalence of multiple genotypes. A recent increase in detection rate in less reported recombinant genotypes such as GII.P12/GII.3 and GII.P7/GII.6 among adult population, together with the discovery of multiple gene clustering in the ORF1-ORF2 junction of both genotypes calls for a continuing close monitoring on NoV GII genotypes in case of potential local outbreaks.

## Additional file

**Additional file 1.** GenBank accession numbers of sequences for strains of interest.

## Data Availability

All data involved in this study is available upon reasonable request made to the corresponding author.
