# Supplementary material for: Genotypic prevalence of norovirus GII in gastroenteritis outpatients in Shanghai from 2016 to 2018
Source: Gut Pathog. 2019 Jul 26;11:40. doi: 10.1186/s13099-019-0321-x (PMC6660925; doi:10.1186/s13099-019-0321-x)
Supplement: Supplementary file 1 — Additional file 1. GenBank accession numbers of sequences for strains of interest. [file 13099_2019_321_MOESM1_ESM.docx]

Table S1 GenBank accession numbers of sequences for strains of interest

| Sample code in this study | GenBank Accession number |
| --- | --- |
| 201609-XH784 | MK779279 |
| 201610-QP649 | MK779280 |
| 201711_FX078 | MK779281 |
| 201711_JA366 | MK779282 |
| 201711_ZB626 | MK779283 |
| 201712_PT238K | MK779284 |
| 201712_PT535 | MK779285 |
| 201801_PT016K | MK779286 |
| 201801_PT239 | MK779287 |
| 201802_JA234 | MK779288 |
| 201810_MH437 | MK779289 |
| 201810_MH615 | MK779290 |
| 201810-CM672 | MK779291 |
| 201810-QP001 | MK779292 |
| 201810-SJ030 | MK779293 |
| 201811-BS219 | MK779294 |
| 201811-FX051K | MK779295 |
| 201811-JA652 | MK779296 |
| 201811-JD083K | MK779297 |
| 201811-QP278 | MK779298 |
| 201812-CN583K | MK779299 |
| 201812-HK511F | MK779300 |
| 201812-JA970 | MK779301 |
| 201812-MH039 | MK779302 |
| 201812-QP455 | MK779303 |
| 201812-SJ511 | MK779304 |
| 201611_PT250K | MK789447 |
| 201612_BS655 | MK789448 |
| 201710_PT308 | MK789449 |
| 201711_BS053 | MK789450 |
| 201801_PT005K | MK789451 |
| 201802_MH063 | MK789452 |
| 201806_HK861F | MK789453 |
| 201807_PD271 | MK789454 |
| 201807_PD491 | MK789455 |
| 201808_MH465 | MK789456 |
| 201808_PD563 | MK789457 |
| 201808_QP090 | MK789458 |
| 201808_QP462 | MK789459 |
| 201809_CN032K | MK789460 |
| 201809_HK211F | MK789461 |
| 201810_XH422 | MK789462 |
| 201811_CM854 | MK789463 |
